# Molecular determinants of improved cathepsin B inhibition by new cystatins obtained by DNA shuffling

**DOI:** 10.1186/1472-6807-10-30

**Published:** 2010-09-30

**Authors:** Napoleão F Valadares, Márcia Dellamano, Andrea Soares-Costa, Flávio Henrique-Silva, Richard C Garratt

**Affiliations:** 1 Center for Structural Molecular Biotechnology, Department of Physics and Informatics, Physics Institute of São Carlos, University of São Paulo, Av. Trabalhador são-carlense 400, 13560-970, São Carlos-SP, Brazil; 2 Laboratory of Molecular Biology, Department of Genetic and Evolution, Federal University of São Carlos, Rodovia Washington Luis km 235, CEP 13565-905, São Carlos-SP, Brazil

## Abstract

**Background:**

Cystatins are inhibitors of cysteine proteases. The majority are only weak inhibitors of human cathepsin B, which has been associated with cancer, Alzheimer's disease and arthritis.

**Results:**

Starting from the sequences of oryzacystatin-1 and canecystatin-1, a shuffling library was designed and a hybrid clone obtained, which presented higher inhibitory activity towards cathepsin B. This clone presented two unanticipated point mutations as well as an N-terminal deletion. Reversing each point mutation independently or both simultaneously abolishes the inhibitory activity towards cathepsin B. Homology modeling together with experimental studies of the reverse mutants revealed the likely molecular determinants of the improved inhibitory activity to be related to decreased protein stability.

**Conclusion:**

A combination of experimental approaches including gene shuffling, enzyme assays and reverse mutation allied to molecular modeling has shed light upon the unexpected inhibitory properties of certain cystatin mutants against Cathepsin B. We conclude that mutations disrupting the hydrophobic core of phytocystatins increase the flexibility of the N-terminus, leading to an increase in inhibitory activity. Such mutations need not affect the inhibitory site directly but may be observed distant from it and manifest their effects via an uncoupling of its three components as a result of increased protein flexibility.

## Background

The human cathepsins B and L are cysteine proteases of the papain subfamily, which primarily function as endopeptidases within endolysosomal compartments. Causal roles for cathepsins in cancer have been demonstrated by pharmacological and genetic techniques [1], and different mechanisms were shown to increase the expression of cathepsins B and L in tumours [2]. Furthermore, given the involvement of cathepsin B in neurobiological functions and neurodegenerative disease [3], tumor progression and arthritis [2], a better understanding of its function at the molecular level and of the mechanisms of cathepsin inhibition is desirable.

Cystatins are a group of cysteine protease inhibitors that have been identified in vertebrates, invertebrates, and plants. Plant cystatins, also known as phytocystatins, are proteins characterized by the absence of disulfide bonds and putative glycosilation sites, which cluster in a major evolutionary tree branch of the cystatin superfamily of proteins [4]. In plants, phytocystatins regulate endogenous proteolytic activities, also having a role in improving defense mechanisms against insects and pathogens [5]. Recent studies have characterized sugarcane cystatins [6-8], proteins that have a role in resistance to pathogenic attacks towards sugarcane (*Saccharum officinarum*), a crop extensively cultivated in Brazil due to its economic implications as a renewable energy source [9].

The best studied phytocystatin is oryzacystatin-1 from rice, whose fold can be described as a five-stranded antiparallel β-sheet wrapped around a central helix [10], being stabilized by a hydrophobic cluster formed between the two which contains a specific LARFAV-like conserved sequence present only in phytocystatins [4]. Cystatins use three structural elements to interact and inhibit cysteine proteases, two loops together with the N-terminal region. Both loops physically interact with the active site of the cysteine protease, the first through its QXVXG motif (residues Q53 to G57 in oryzacystain-1) and the second via residues P83 and W84. The N-terminal region does not directly interact with the active site, but makes extensive contacts with the protease, playing an important role in the binding process [10-12].

Here, we describe the use of DNA shuffling to create a new hybrid cystatin with improved cathepsin B inhibitory activity, obtained through the recombination of canecystatin-1 and oryzacystatin-1. The activity and physicochemical properties of three other mutants obtained through the reversion of point mutations observed in this hybrid, as well an N-terminally deleted version of oryzacystatin, were also determined. Analysis of molecular models of these recombinant proteins was used to explain the molecular determinants of their activities.

## Methods

### DNA shuffling library construction

The method used involves the fragmentation of genes with similar DNA sequences using DNase I to generate a pool of random DNA fragments. These fragments were reassembled into a full-length gene by repeated cycles of annealing in the presence of DNA polymerase. The fragments prime on each other based on sequence homology, and recombination occurs when fragments from one gene anneal to fragments from the other, causing a template switch.

### Gene Selection

The choice of specific genes encoding counterpart cysteine protease inhibitors in sugarcane (CaneCPI-1, [GenBank:AY119689]) and rice (oryzacystatin I, [GenBank:U54702]) was based on the similarity of their DNA sequences (56%).

### Substrate Preparation

The principle of DNA shuffling is recombining distinct genes that present high similarity in their DNA sequence. In our case, the selected genes CaneCPI-1 and OC-I were used in the construction of the shuffling library. The substrates used for the shuffling reactions were PCR products obtained from the amplification of the CaneCPI-1 and OC-I genes using the pET28aCaneCPI-1 [6] and pET28OC-I [13] plasmids respectively, as templates. For CaneCPI-1 amplification by PCR the following primer sequences were employed: CaneCPI-1F (5' TCGAAGGTCGTCATATGATGGCCGAGGCAC 3´) and T7 terminator (5' TAGTTATTGCTCAGCGGTGG 3'). In the case of the OC-I gene the primer T7 promoter ('5 TAATACGACTCACTATAGGG 3') together with the T7 terminator primer were used. Free primers from the PCR product were removed by Wizard PCR (Promega).

### DNAse I Digestion

About 4 μg of amplification product (DNA substrate) were digested with 0.15 unit of DNAse I (10U/μl) in 100 μl of buffer containing 50 mM Tris-HCl, pH 7.4, 1 mM MnCl_2_, for 10-20 min at room temperature. Fragments of 40-120 bp were recovered from 2% low melting point agarose gels by electrophoresis using the gel Kit QIAEX II Agarose Gel Extraction (QIAGEN) and ethanol precipitated.

### PCR without primer

The recovered fragments of 40-120 bp obtained from DNase I digestion were used in the reaction of recombination. The first extension was performed using 5 μl of each purified fragment and re-suspended in 20 μl of PCR mixture containing 0.2 mM each dNTPs, 1.5 mM MgCl2, 0.1 μl *Taq DNA Polymerase *(5U/μl), 0.1 μl *Pfu Turbo DNA Polymerase *(2.5 U/μl) and 2 μl *Taq *buffer 10×. This solution was submitted to a round of extension of 40 cycles at 95°C/30 sec, 50°C/30 sec, 72°C/2 min + 2 sec/cycle.

### PCR with Primers

8 μl of recombination product obtained from PCR without primers were used in 100 μl of PCR mixture with 0.2 mM dNTPs, 10 μl of amplification buffer (10×), 3 μl MgCl_2 _(50 mM), 2 μl first OC-I forward primer (10 pmol/μl) and CaneCPI-1 reverse primer (10 pmol/μl), 2.5 U of *Taq DNA Polymerase*. The conditions for amplification were: 1× [94°C/1 min], 35× [94°C/1 min, 47°C/1 min and 72°C/1.5 min] and 1× [72°C/5 min]. The amplification product was submitted to analysis by agarose gel electrophoresis and amplified DNA was purified from the gel using the QIAEX II gel extraction kit (QIAGEN).

### Cloning and Analysis

The plasmid pET28a was cut with *Eco *RI and *Nde *I and dephosphorylated with shrimp alkaline phosphatase (SAP) in 5 μl buffer containing 200 mM Tris pH 8.0 and 100 mM MgCl2; 1 μl SAP (1U/μl) and water to 50 μl for an incubation of 1 hour at 37°C. The inactivation of SAP was performed at 70°C for 20 min. This solution was precipitated with ethanol and re-suspended in water to a final volume of 30 μl. The products of final amplification were digested with the restriction enzymes *Eco *RI and *Nde *I and ligated in the dephosphorylated pET28a plasmid. The ligation reaction was used to transform *E. coli *Rosetta (DE3), for expression of the hybrid proteins.

About 2000 clones were sequenced by the dideoxy method [14] using an ABI Prism 377 (Applied Biosystems). The sequences obtained were then analyzed using BLAST alignments and the software Multalin http://bioinfo.genotoul.fr/multalin/multalin.html in order to find a clone resulting from recombination. Based on the recombinants obtained several clones were selected and submitted to expression analysis and subsequent inhibitory activity assays.

### Site-directed mutagenesis

Site-directed mutagenesis was performed using the Gene Tailor™Site Directed Mutagenesis System (Invitrogen). The pET28a encoding the mutant A10 gene was used as template DNA for the construction of the reverse mutant 1 (T30I) and mutant 2 (Q97L). The DNA corresponding to mutant 2 was used as a template for the construction of reverse mutant-3 (the double mutant) using the mutant-1 primers to allow for both mutations. The primer sequences were as follows: mutant-1 forward, 5'-GACCTCGAGGCCATCGAGCTCGCGCGC-3'; mutant-1 reverse, 5'-CTTGTCCTTGCTGAGCTCCGG-3'; mutant-2 forward, 5'-AACTTCAAGCAGCTGCAGAGCTTCAG-3'; mutant-2 reverse, 5'-CCACACCCTCTTGAAGTTCGTC-3'. PCR products were analyzed on agarose gels to confirm the presence of a product of the correct molecular weight and all plasmids were sequenced.

The recombinant cystatins were expressed in *E. coli *Rosetta (DE3) carrying the appropriate pET28a vector, and purified as previously described [6,8].

### Expression of recombinant cystatins and mutants

Two clones of the shuffling library were selected for this study. One of these, here termed OC-I NΔ, was a pure oryzacystatin clone which presented a seven-residue N-terminal deletion. The second was a hybrid clone containing two mutations besides the N-terminal deletion (clone A10). These clones were selected for expression and inhibition assays together with OC-1, CaneCPI-1, CaneCPI-4, and the mutant 1, 2, and 3. The corresponding constructs were used to transform competent strains of *E. coli *Rosetta (DE3) with calcium chloride. The transformed cells were cultivated at 37°C under agitation in selective medium containing kanamycin (25 mg/mL) until they reached an optical density (O.D.) of 0.6, at 600 nm, when protein expression was induced by the addition of IPTG to a final concentration of 0.4 mM. Aliquots were taken for up to 4 h (at 1 hour intervals) after induction and the cell extract was analyzed on SDS-PAGE 15% [15]. After induction, the cells were collected, centrifuged, and subjected to a solubility test. To this end, the cells were suspended in suspension buffer containing 10 mM Tris-HCl, 100 mM NaCl, 50 mM NaH_2_PO_4_, pH 8.0 and subjected to lyses by sonication five times for 1 min at 30 s intervals. The lysed cells were centrifuged at 13,000 g and 4°C for 10 min, and the supernatant and precipitate analyzed on SDS-PAGE 15% [15].

### Purification of the recombinant proteins

The fraction containing the soluble proteins was purified from the supernatant using a nickel affinity column, Ni-NTA superflow (Qiagen). The column was equilibrated and washed with two column volumes of suspension buffer and after sample application the proteins were eluted with increasing imidazol concentrations (10, 25, 50, 75, 100, and 250 mM). The purified proteins were analyzed on SDS-PAGE 15%. The fractions containing the purified proteins were dialyzed using MWCO: 3 membranes (Spectrum Laboratories) and the concentrations determined by Bradford's method [16].

### Enzyme inhibition activity

The inhibitory activity of the recombinant cystatins was measured against human cathepsins B and L (Calbiochem) using the fluorogenic substrate Z-Phe-Arg-MCA (Calbiochem) as previously described [8]. Briefly, human cathepsins B and L (0.3 nM) were individually incubated for 5 min at 37°C with different inhibitors CaneCPI-1, OC-I, OC-I NΔ, A10, mutant 1, mutant 2 and mutant 3 in a buffer containing 100 mM sodium acetate pH 5.5, 2.5 mM DTT. The concentration range of each inhibitor is presented in Additional file 1. The substrate Z-Phe-Arg-MCA (0.01 mM) was added and the residual hydrolytic activity was monitored using a Hitachi F-2500 spectrofluorometer (λ_ex _= 380 nm and λ_em _= 460 nm). All experiments were carried out in triplicate and the results used to determine *K*_i(app) _by non linear regression using the GraFit program [17]. The equilibrium inhibition constant (*K*_i_) of the enzyme inhibitor complex was subsequently calculated using Morrison's procedure [18,19].

### Molecular Modeling

The amino acid sequences of oryzacystatin-1 and human stefin B were retrieved from Swiss-Prot (accession numbers [Swiss-Prot:P09229] and [Swiss-Prot:P04080], respectively) and the three-dimensional structures were obtained from the protein databank (1EQK and 1STF, respectively). The sequences were aligned using CLUSTALX http://www.clustal.org/download/current/ and the result manually adjusted based on structural superposition. The sequences of the cystatins were then aligned to this template.

Comparative molecular models corresponding to each of these alignments were obtained using the program MODELLER 9v8 [20]. A series of different models were generated and their quality evaluated by the MODELLER pseudo-energy term and its DOPE score [21]. The models were also subjected to independent evaluation by the programs VERIFY 3D [22] and WHATIF [23], and a representative model for structural analysis was selected.

## Results and Discussion

A total of two thousand clones were sequenced from the shuffling library. Amino acid sequence analyses were made in order to find a hybrid formed by the CaneCPI-I and OC-I proteins. It was expected that the DNA encoding two distinct but similar proteins would form a heteroduplex hybrid, but in practice most of the analyzed clones in the library were homoduplex. Approximately 50% and 25% of clones were identical to OC-I and CaneCPI-1, respectively. A further 20% corresponded to OC-I with the N-terminal deletion (OC-I NΔ) and the remaining 5% were shuffled, truncated or presented point mutations. From among the many clones OC-I NΔ and A10, the latter belonging to the remaining 5%, were selected to be of potential interest.

**Figure 1:**
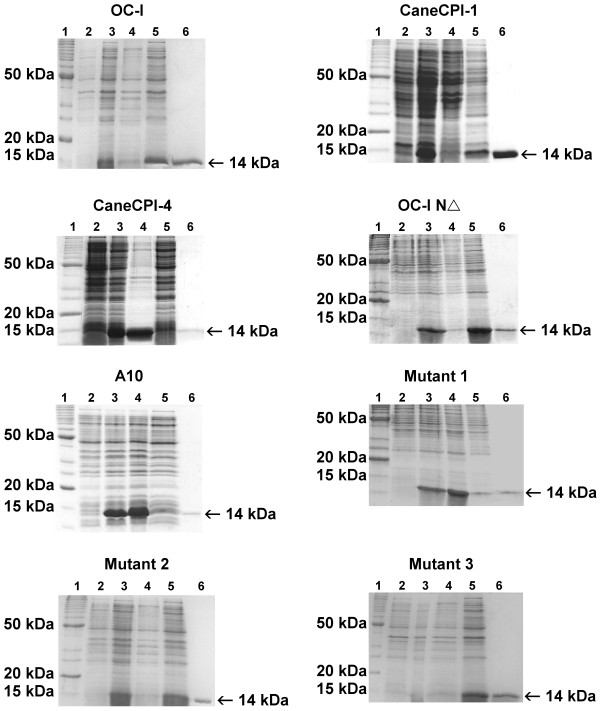
**Expression and purification of phytocystatins and mutants**. SDS-PAGE 15% stained with Coomassie blue showing OC-I, CaneCPI-1, CaneCPI-4, OC-I NΔ, A10, mutant 1, mutant 2 and mutant 3. Samples were collected and analyzed in SDS-PAGE 15%. In (1) molecular mass marker; (2) *E. coli *Rosetta (DE3) cell extract before and (3) after IPTG induction, (4) insoluble and (5) soluble fractions after disruption of induced cells from *E. coli *Rosetta (DE3), and (6) purified recombinant protein after elution with 250 mM imidazole from a nickel column.

Expression and purification assays for OC-I, CaneCPI-1, CaneCPI-4, OC-I NΔ, A10, mutant 1, mutant 2 and mutant 3 was performed and analyzed in a Coomassie blue stained SDS-PAGE (Figure 1). This analysis revealed the presence of the His-tagged proteins of expected sizes for the induced clones, the insoluble and soluble fractions, and the purified recombinant protein. Most of the recombinant proteins were in their soluble form and could be purified directly by affinity chromatography on a nickel column using 250 mM imidazole. Even CaneCPI-4, A10 and mutant 1, which presented high amounts of protein in the insoluble fraction, could be purified from the supernatants. The amounts of pure recombinant proteins obtained after a single step of purification were sufficient for performing activity tests.

The cysteine peptidases cathepsin L and B were assayed in the presence of the recombinant cystatins. Their inhibitory activity was assessed in a fluorometric assay using Z-Phe-Arg-MCA as substrate for which cathepsin L and B present *K*_M _values of 2 μM and 23.4 μM, respectively [24]. The residual hydrolytic activity of the enzyme was measured after pre-incubation for 5 minutes with the inhibitors at different concentrations. The resulting *K*_i _values are shown in Table 1.

**Table 1:** Inhibition of cathepsins B and L by cystatins.^a^

Cystatin	*K*_i_	
	
	Cathepsin B	Cathepsin L
Oryzacystatin-1	78.5 nM	0.73 nM
Canecystatin-1	87.6 nM	0.10 nM
Canecystatin-4^b^	0.58 nM	0.0035 nM
Oryzacystatin-1 N-terminal deletion (NΔ)	NI	5.66 nM
Clone A10	11.2 nM	1.63 nM
Reverse Mutant 1 (T30I)	NI	1.93 nM
Reverse Mutant 2 (Q97L)	NI	11.7 nM
Reverse Mutant 3 (T30I, Q97L)	NI	12.5 nM

^a ^NI indicates no inhibitory activity observed at the highest concentration of the inhibitor employed.

^b ^data previously published [8].

Two clones in particular (OC-I NΔ and A10) presented interesting profiles in terms of enzyme inhibition. The majority of cystatins, such as oryzacystatin-1 (from which OC-I NΔ was derived) and canecystatin-1, bind more tightly to cathepsin L than to cathepsin B, and typically show at least one order of magnitude difference in terms of *K*_i_. OC-I NΔ, on the other hand, presented no activity towards Cathepsin B whilst still retaining moderate activity towards cathepsin L. A completely different profile was shown by A10 which presented comparable inhibition of both enzymes due to an increased activity towards Cathepsin B (*K*_i _= 11.21 nM, see Table 1).

**Figure 2:**
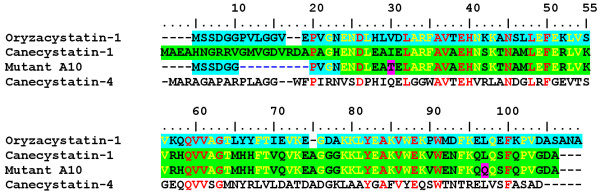
**Sequence alignment of relevant cystatins**. Sequence alignment between oryzacystain-1 (cyan), canecystatin-1 (green), A10 and canecystatin-4. Conserved amino acids are show in red. Residues conserved in all sequences except canecystatin-4 are coloured in yellow. In the case of the clone A10 the residues are shaded according to the cystatin from which they were derived with point mutations shaded in purple, and the N-terminal deletion from residues 11 to 19 is shown as a blue gap. The clone OC-I NΔ has the sequence of oryzacystatin-1, but with the same N-terminal deletion as the clone A10.

Four differences can be noted between A10 and the original canecystatin-1 from which it is largely derived. Firstly, the N-terminal region of A10 comes from oryzacystatin-1 and not canecystatin-1, a result of the gene shuffling process itself. Secondly, this region has suffered a 7 amino acid deletion (Figure 2). Finally, A10 has acquired two unexpected point mutations affecting hydrophobic residues of the protein core, I30T at the beginning of the α-helix and L97Q in strand β5 (residue numbers follow those of canecystatin-1 throughout the text unless otherwise stated, see Figure 2).

Table 1 also shows data on enzyme inhibition by mutants in which these specific peculiarities of A10 were individually dissected. A deletion mutant of oryzacystatin-1 which reproduces the effect of the loss of seven residues towards the N-terminus of A10, retained nanomolar activity towards cathepsin L, but lost all of its activity towards Cathepsin B. This is consistent with the current model for cathepsin B inhibition by cystatins in which initial binding of the N-terminal region precedes a conformational change to the occluding loop [25,26]. Changes to this region are known to significantly affect inhibitory activity towards cathepsin B, and the residues lost as a consequence of the deletion have been identified as important for protease binding [12]. It is therefore not entirely unexpected that the two reverse mutants of A10 (T30I and Q97L), which also present the 7-residue deletion, were also unable to significantly inhibit Cathepsin B. On the other hand both retained their ability to inhibit Cathepsin L at the nMolar level, indicative of correct folding. What is more surprising is the ability of A10 itself to inhibit cathepsin B at all. The accumulation of the two mutations together is somehow able to overcome the deleterious effect of the N-terminal deletion and to turn A10 into a nMolar inhibitor of cathepsin B.

### 3.3. Sequences and molecular homology models analysis

**Figure 3:**
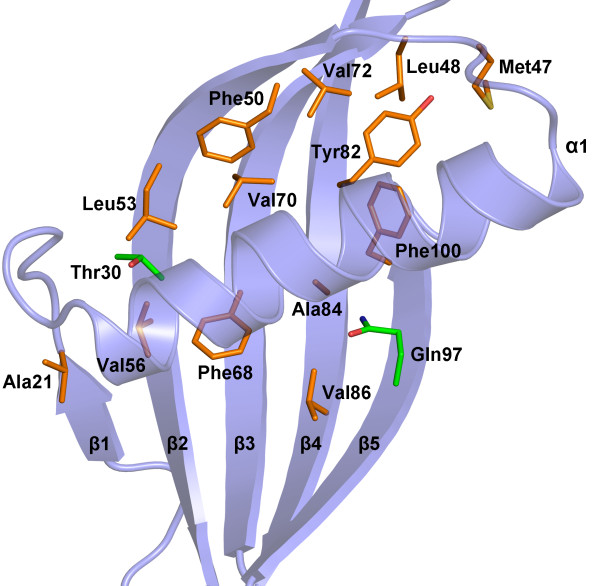
**The hydrophobic core residues arising from the anti-parallel β-sheet of clone A10**. The transparent cartoon shows the three-dimensional fold of the protein in blue, and the residues participating in the hydrophobic core are colored in orange. Threonine 30 (from the α-helix) and glutamine 97 are coloured in green.

A homology model for the canecystatin-1 structure shows that, unlike a similar model for clone A10, it preserves the hydrophobic core seen in oryzacystatin-1 [10]. This is located at the interface between the five-stranded anti-parallel β-sheet and the single α-helix (Figure 3). In the β-sheet of canecystatin-1, the residues involved in this interface are M47, L48, F50, L53, V56 (in strand β2), F68, V70, V72 (strand β3), Y82, A84, V86 (strand β4) and L97, F100 (strand β5). The clone A10 presents a glutamine residue instead of a leucine in the position 97 (Figure 3). Close to the N-terminus of the helix there is a small strand (β1) which interacts with β2 via both main chain hydrogen-bonds as well as hydrophobic contacts involving A21 and V56, the latter on strand β2. The helix residues most obviously involved in the hydrophobic core are I30 and the residues of the conserved LARFAV sequence. The remarkable conservation of this motif among phytocystatins has been emphasized previously, but the authors were unable to attribute to it a specific functional role [27]. We propose that the role of this motif is to provide ideal complementarity to the hydrophobic residues in the β-sheet of the phytocystatins, essential for stabilizing the tertiary structure.

The three active site segments of phytocystatins which directly interact with the binding pocket of the enzyme have been proposed based on the complex formed between stefin B and papain [10,11]. The first interacting loop corresponds to canecystatin-1 residues 59-63, presenting the highly conserved sequence QVVAG, which is identical in oryzacystatin-1 and A10. The second binding loop includes V90 and W91 (P 83 and W84 in oryzacystatin-1) and the third interaction site is formed by the N-terminal region.

These three regions form the classical interaction surface between cystatins and cysteine proteinases. However, Cathepsin B, different from Cathepsin L and most other cysteine proteinases, possesses a large insertion (the occluding loop) which covers part of the binding pocket thus impeding the simultaneous entry of all three elements of the inhibitor's active site. The observed binding of some cystatins to Cathepsin B is explained by a two-step mechanism in which the initial binding of the N-terminal region leads to subsequent displacement of the occluding loop generating an effective binding mode [25,26]. Furthermore, recent studies employing single mutations at positively selected amino acid sites confirm the functional importance of the N-terminal region of phytocystatins [27,28]. Here we show that the N-terminal deletion mutant and the double reverse mutant, both of which present the 7 residue deletion near the N-terminus, are unable to inhibit cathepsin B. On the other hand the two point mutations which restore activity to A10 are located distant from the active site loops and therefore must influence activity via an indirect effect.

**Figure 4:**
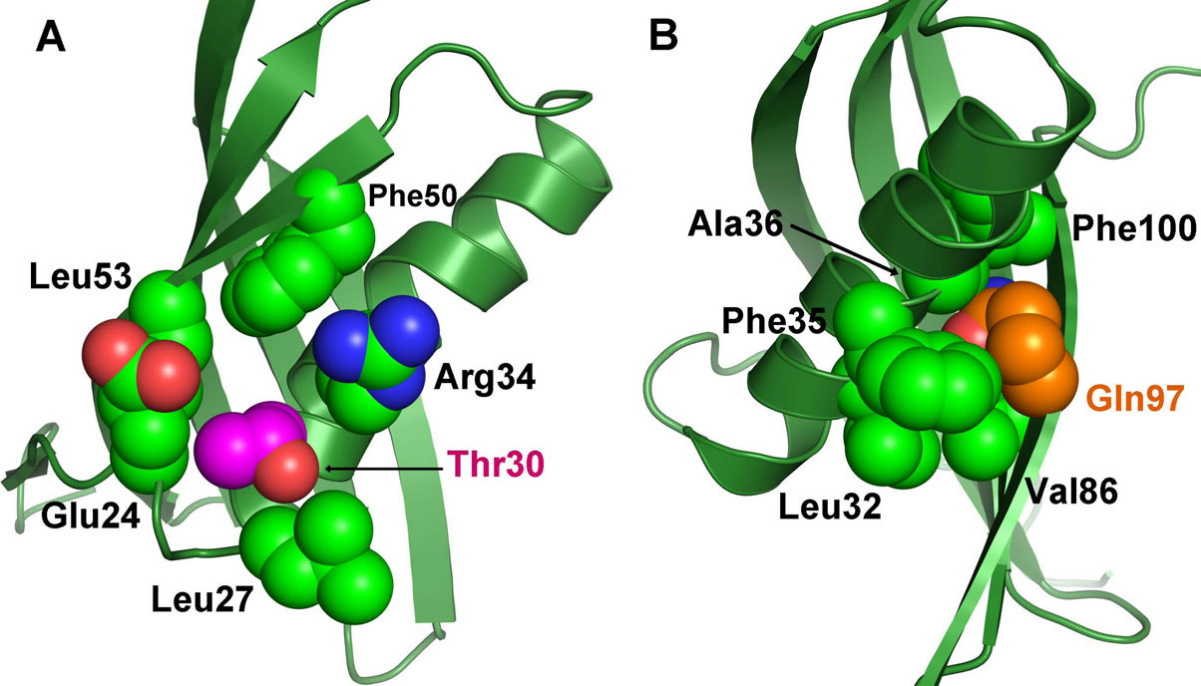
**Homology model of clone A10 showing the residues correspondent to the point mutations**. Interactions of (A) threonine 30 (magenta) and (B) glutamine 97 (orange). Interacting residues are labeled and shown as spheres. It is likely that the mutations would significantly destabilize the hydrophobic core leading to a conformation different to that shown in the figure.

As depicted in Figure 4A, the first mutation (I30T) is located at the beginning of the α-helix where it would be expected to destabilize the hydrophobic cluster formed by residues F50, L53, I30 the aliphatic portion of R34 and the loop connecting the N-terminus to the α-helix (Additional file 2). The second mutation (L97Q) appears yet more significant and perturbs the opposite side of the hydrophobic core formed by L32, F35, A36, V86, F100 and L97 (Figure 4B). We suggest that these mutations would significantly destabilize the hydrophobic contacts which hold the helix against the β-sheet, thus leading to its complete or partial release. This release would have the effect of decoupling two components of the inhibitor active site; the N-terminal region on the one hand and the remaining two loops on the other.

The increased flexibility of the N-terminal region in the A10 mutant may allow it to regain its role in the initial binding to the enzyme as the first step in the well established two step mechanism. Alternatively, the uncoupling of the three components of the inhibitor active site may reduce steric hindrance and facilitate direct binding by the QVVAG and VW loops to the catalytic site.

It is noteworthy that the observation of three-dimensional domain swapping within the cystatin family involves exactly the type of structural rearrangement that we are proposing here [29]. This allows cystatins to self assemble into different oligomeric states [30,31] and even form amyloid fibrils [32,33]. Thus it would seem that perturbation of the hydrophobic contacts between these secondary structure elements could readily lead to destabilization of this interface. This hypothesis is further supported by the observation that A10 is much less soluble than the parent molecules and tends to aggregate in inclusion bodies when heterologously expressed (Figure 1), consistent with the exposure or partial exposure of its hydrophobic core. The single reverse mutants show intermediate solubilities, with the T30I mutant (which retains the glutamine at position 97) being the less soluble of the two. However, the inhibition data on these mutants demonstrates that both mutations are necessary for the increased activity of A10 towards cathepsin B.

Although A10 still presents a lower activity towards cathepsin B than other natural cystatins such as its endogenous inhibitor cystatin C, the hypothesis raised here presents a rational basis which might be exploitable in the development of tighter binding cathepsin B inhibitors. In this context it is worth mentioning that sugar cane expresses several such inhibitors besides canecystatin-1. For example canecystatin-4 has been reported to have an affinity comparable to that of cystatin C [8,25]. Figure 2 shows that canecystatin-4 also presents interesting variations to some of the hydrophobic residues at the interface between the helix and the β-sheet, including a glutamine at position 30 (corresponding to one of the positions mutated in A10) and glycines at positions 47 and 56, which decrease the volume of the hydrophobic core. Furthermore it has been reported that canecystatin-4 tends to aggregate more readily than canecystatin-1 [8].

In summary, it is hoped that the methodology and structural insights presented here can be useful in the design of more potent and specific cathepsin inhibitors, as well as contributing to the rationalization of the activity of already characterized cystatins. Specifically, mutations outside the N-terminal region which lead to an altered mobility may be an interesting alternative approach compared with modifying the region itself.

## Conclusions

A combination of experimental approaches including gene shuffling, enzyme assays and reverse mutation has been used to better understand the inhibitory properties of cystatin mutants against Cathepsin B. Molecular modeling of the mutant enzymes suggests that disruption of the hydrophobic core may lead to an increase in the flexibility of the N-terminus, and consequently an increase in inhibitory activity. Such mutations need not affect the inhibitory site directly, but may be observed distant from it and manifest their effects via an uncoupling of its three components as a result of increased protein flexibility.

## Authors' contributions

All authors read and revised the manuscript and approved the final version. MD, ASC and FHS planned and carried out the molecular genetic studies. NFV and RCG planned and performed the structural analysis and wrote the manuscript.

## Supplementary Material

Additional file 1 **Additional Table S1**. Concentrations of the different inhibitors used in the enzyme inhibition assays for cathepsins B and L. Click here for file

Additional file 2 **Video: Molecular modeling of the Canecystatin-1**. The hydrophobic cluster formed by residues Phe50, Leu53, Ile30 the aliphatic portion of Arg34 and the loop connecting the N-terminus to the α-helix. Ile30 is coloured in magenta, and is mutated to threonine in the clone A10. Click here for file
