# Supplementary material for: Molecular determinants of improved cathepsin B inhibition by new cystatins obtained by DNA shuffling
Source: BMC Struct Biol. 2010 Sep 30;10:30. doi: 10.1186/1472-6807-10-30 (PMC2959088; doi:10.1186/1472-6807-10-30)
Supplement: Additional file 1 — Additional Table S1. Concentrations of the different inhibitors used in the enzyme inhibition assays for cathepsins B and L. [file 1472-6807-10-30-S1.DOC]

**Molecular determinants of improved cathepsin B inhibition by new cystatins obtained by DNA shuffling.**

Napoleão F. Valadares^1*^, Márcia Dellamano^2*^, Andrea Soares-Costa^2^, Flávio Henrique-Silva^2§^ and Richard C. Garratt^1§^

1 - Center for Structural Molecular Biotechnology, Department of Physics and Informatics, Physics Institute of São Carlos, University of São Paulo, Av. Trabalhador são-carlense 400, 13560-970, São Carlos-SP, Brazil.

2 - Laboratory of Molecular Biology, Department of Genetic and Evolution, Federal University of São Carlos, Rodovia Washington Luis km 235, CEP 13565-905, São Carlos-SP, Brazil

* These authors contributed equally to this work

^§^Corresponding authors

E-mail addresses:

NFV: [napo@ifsc.usp.br](mailto:napo@ifsc.usp.br)

MD: [mdellamano@yahoo.com.br](mailto:mdellamano@yahoo.com.br)

ASC: [pascosta@ig.com.br](mailto:pascosta@ig.com.br)

FHS: [dfhs@power.ufscar.br](mailto:dfhs@power.ufscar.br)

RCG: [richard@ifsc.usp.br](mailto:richard@ifsc.usp.br)

**Additional Material**

**Table S1**. Concentrations of the different inhibitors used in the enzyme inhibition assays for cathepsins B and L.

| Cystatin | Cystatin Concentrations | |
| --- | --- | --- |
|  | Cathepsin B | Cathepsin L |
| Oryzacystatin-1 | 25, 50, 75, 100, 125 and 150 nM | 2, 4, 6, 8 and 10 nM |
| Canecystatin-1 | 25, 50, 75, 100, 125 and 150 nM | 0.2, 0.4, 0.6, 0.8 and 1 nM |
| Canecystatin-4^b^ | 0.2, 0.4, 0.6, 0.8 and 1 nM | 0.01, 0.02, 0.03, 0.04 and 0.05 nM |
| Oryzacystatin-1  N-terminal deletion (NΔ) | 25, 50, 75, 100, 125 and 150 nM | 8, 16, 24, 32 and 40 nM |
| Clone A10 | 8, 16, 24, 32 and 40 nM | 4, 8, 12, 16 and 18 nM |
| Reverse Mutant 1 (T30I) | 25, 50, 75, 100, 125 and 150 nM | 5, 10, 15, 20 and 25 nM |
| Reverse Mutant 2 (Q97L) | 25, 50, 75, 100, 125 and 150 nM | 25, 50, 75, 100, 125 and 150 nM |
| Reverse Mutant 3 (T30I,Q97L) | 25, 50, 75, 100, 125 and 150 nM | 25, 50, 75, 100, 125 and 150 nM |

^b^ data previously published [8].
