# Supplementary figures and images for: Molecular determinants of improved cathepsin B inhibition by new cystatins obtained by DNA shuffling
Source: BMC Struct Biol. 2010 Sep 30;10:30. doi: 10.1186/1472-6807-10-30 (PMC2959088; doi:10.1186/1472-6807-10-30)

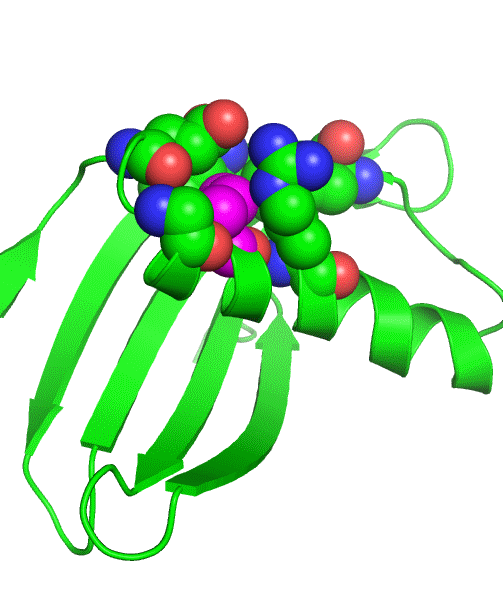

Supplement: Additional file 2 — Video: Molecular modeling of the Canecystatin-1. The hydrophobic cluster formed by residues Phe50, Leu53, Ile30 the aliphatic portion of Arg34 and the loop connecting the N-terminus to the α-helix. Ile30 is coloured in magenta, and is mutated to threonine in the clone A10. [file 1472-6807-10-30-S2.GIF]
